# Optimal Detection of Latent Mycobacterium tuberculosis Infection by Combined Heparin-Binding Hemagglutinin (HBHA) and Early Secreted Antigenic Target 6 (ESAT-6) Whole-Blood Interferon Gamma Release Assays

**DOI:** 10.1128/jcm.02443-21

**Published:** 2022-04-18

**Authors:** V. Dirix, N. Dauby, M. Hites, E. Watelet, A. Van Praet, A. Godefroid, E. Petit, M. Singh, C. Locht, F. Mascart, V. Corbière

**Affiliations:** a Laboratory of Vaccinology and Mucosal Immunity, Université Libre de Bruxelles, Brussels, Belgium; b Department of Infectious Diseases, Centre Hospitalier Universitaire Saint-Pierre, Université Libre de Bruxelles, Brussels, Belgium; c Institute for Medical Immunology, Université Libre de Bruxelles, Brussels, Belgium; d Department of Infectious and Tropical Diseases, Hôpital Erasme, Université Libre de Bruxelles, Brussels, Belgium; e Department of Pneumology, Clinique St. Anne/St. Remi-CHIREC, Brussels, Belgium; f Université de Lille, CNRS, INSERM, CHU Lille, Institut Pasteur de Lille, U-1019, UMR8204, Center for Infection and Immunity of Lille, Lille, France; g Lionex Diagnostics and Therapeutics, Braunschweig, Germany; University of Manitoba

**Keywords:** latent tuberculosis infection, interferon gamma release assay, whole blood, heparin-binding hemagglutinin, early secreted antigenic target 6

## Abstract

Optimal detection of latent tuberculosis (TB) infection (LTBI) remains a challenge, although it is essential to reach the goal of TB elimination. Our objective was to develop and clinically evaluate a user-friendly, 24-h, whole-blood (WB) interferon gamma (IFN-γ) release assay (IGRA) improving the detection of LTBI, compared to available tests. One milliliter of blood was divided into four aliquots and *in vitro* stimulated for 24 h with two different stage-specific mycobacterial antigens, i.e., heparin-binding hemagglutinin (HBHA) and early secreted antigenic target 6 (ESAT-6), a latency-associated antigen and a bacterial replication-related antigen, respectively, in addition to positive and negative controls. Clinical evaluation was performed on two independent cohorts of carefully selected subjects, i.e., a training cohort of 83 individuals and a validation cohort of 69 individuals. Both cohorts comprised LTBI subjects (asymptomatic people with a positive tuberculin skin test result and potential exposure to TB index cases), patients with active TB (aTB), and noninfected controls. The sensitivity and specificity of the WB-HBHA-IGRA to identify LTBI subjects among asymptomatic individuals were 93%. Combining the results in response to HBHA and ESAT-6 allowed us to identify LTBI subgroups. One group, with IFN-γ responses to HBHA only, was easily differentiated from patients with aTB. The other group, responding to both antigens like the aTB group, is likely at risk to reactivate the infection and should be prioritized for prophylactic anti-TB treatment. The combined WB-IGRA may be offered to clinicians for the selection of LTBI subjects to benefit from prophylactic treatment.

## INTRODUCTION

Tuberculosis (TB) remains a global health problem, with an estimated 10 million new active TB (aTB) cases and 1.5 million deaths in 2020 (1). It is estimated that about one-quarter of the world’s population lives with a latent TB infection (LTBI). These individuals have a lifetime risk of about 5% to 10% to reactivate their infection, leading to progression to aTB disease. This risk is highest early after the infection and in clinical conditions associated with immunodeficiency (2). Because LTBI subjects thus represent an important reservoir of the causative TB agent Mycobacterium tuberculosis, their detection and preventive anti-TB treatment and/or surveillance in countries with both low and high TB burdens are critical for global TB elimination (3).

In the absence of a gold standard (2), the reference test for the detection of LTBI is the tuberculin skin test (TST), which in some countries has been replaced by blood interferon gamma (IFN-γ) release assays (IGRAs). Commercial IGRAs are based on the release of IFN-γ by blood cells in response to *in vitro* stimulation with a mixture of two mycobacterial antigens, namely, early secreted antigenic target 6 (ESAT-6) and culture filtrate protein 10 (CFP-10), or their peptides (2, 4, 5). IGRAs are reported to be more specific than the TST. However, their sensitivity to detect LTBI has been challenged in several studies (6–8). Moreover, neither the TST nor these IGRAs are able to discriminate LTBI from aTB (2). They yield similar predictive values for progression to aTB (9).

We have developed an IGRA on peripheral blood mononuclear cells (PBMC) that is based on the latency-associated antigen heparin-binding hemagglutinin (HBHA) (10), which was found to improve the differential diagnosis between LTBI and aTB (10–13). HBHA is a methylated protein that is expressed by the members of the M. tuberculosis complex and induces IFN-γ responses in LTBI subjects, while these responses are inhibited by CD4^+^ regulatory T lymphocytes during aTB (14). Furthermore, HBHA-IGRAs performed on PBMC are highly sensitive in detecting LTBI, even in subjects with negative commercial IGRA results (10, 15).

In this study, we evaluated the diagnostic performance of a new, user-friendly, 24-h whole-blood (WB)-IGRA, based on separate *in vitro* stimulations with HBHA and ESAT-6, to identify the full spectrum of LTBI subjects in Belgium, a country with a low TB incidence. We demonstrate with two independent cohorts (a training cohort and a validation cohort) of exposed health care workers (HCWs) and household contacts (HHCs) that the combination of WB-HBHA-IGRA and WB-ESAT-6-IGRA allows us to detect LTBI subjects with high sensitivity and to identify two subgroups, likely representing different reactivation risk groups.

## MATERIALS AND METHODS

### Study population.

**FIG 1:**
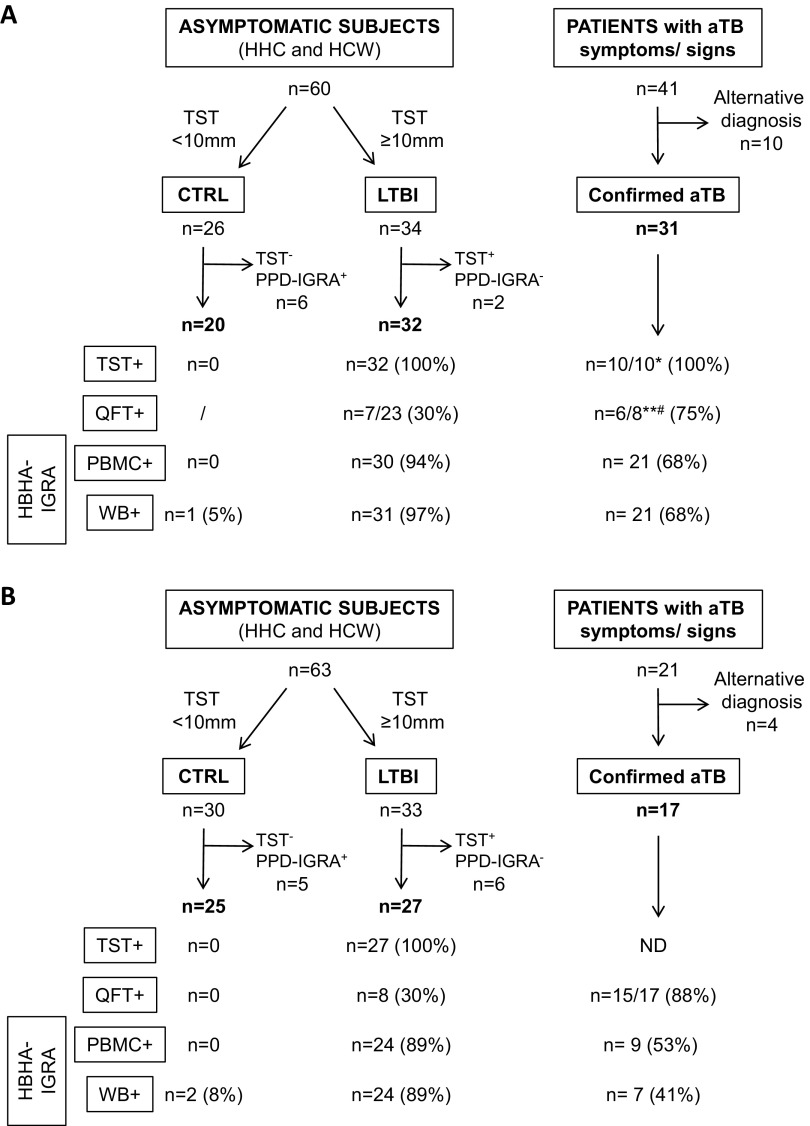
STARD diagram reporting the flow of asymptomatic subjects and aTB patients for evaluation of the HBHA-WB-IGRA to identify LTBI subjects. The WB-HBHA-IGRA was compared to the TST reference recommended in Belgium for LTBI detection, to the QFT, and to the PBMC-HBHA-IGRA. The four tests were performed on noninfected controls (CTRL) and LTBI subjects included in the training cohort (A) and the validation cohort (B). Controls and LTBI subjects who were positive and negative, respectively, with an PPD-IGRA were excluded from the analysis. The criteria for positive TST results were according to the CDC guidelines, and those for positive QFT results were according to the manufacturer’s instructions; for the HBHA-IGRA, positivity was considered when the IFN-γ responses were above the lower value of the gray zone for the defined cutoff value. aTB patients were included for differential diagnosis, and only those with a confirmed diagnosis were retained for the final analysis. *, TST was not done for 21 subjects; **, QFT was not done for 23 subjects; #, 1 indeterminate result.

To ensure the reproducibility of the conclusion, a training or discovery cohort and an independent validation cohort were prospectively enrolled between 2012 and 2015 and in 2016 to 2017, respectively. Healthy subjects, mostly recruited among HCWs and HHCs, were prospectively included in the study on a voluntary basis to enroll both LTBI subjects and uninfected controls with well-defined infectious status. Most LTBI subjects were selected from among HCWs identified as having LTBI by occupational TB screening, most often at least 2 years before inclusion in the study. A TST is performed for HCWs in Belgium every 1 or 2 years and after any known contact with an aTB patient. Most LTBI subjects were thus identified by conversion of their TST, which is not repeated once a person has become positive. A few HCWs suspected to have been recently infected based on TST conversion were also included. In addition, we included HHCs suspected to have LTBI based on positivity of the TST after contact with a patient with aTB (≥10-mm induration, following the CDC recommendations [16]). Clinical examination and chest radiography were performed for all subjects enrolled with LTBI at the time of their diagnosis, to confirm their LTBI status (see Table S1 in the supplemental material). In addition, because various intervals occurred between the identification of a HCW as having LTBI and the blood sampling, a positive homemade purified protein derivative (PPD)-IGRA, performed at the time of blood drawing as described below, was used to confirm the persistence of immune responses to mycobacteria (see Table S1). After applying this PPD-IGRA, 8 LTBI subjects were excluded from the final analysis because of a negative PPD-IGRA result at inclusion, suggesting that they had cured their infection (Fig. 1).

Most uninfected controls were selected from among HCWs with negative TST results. However, 11 controls were excluded from the final analysis because of positive PPD-IGRA results at inclusion (Fig. 1). This strategy was applied to avoid inclusion of potentially infected subjects in the control group, namely, TB resisters with negative TST results in spite of exposure to M. tuberculosis and other specific immune responses (17) or individuals who might have been infected since their last TST.

Patients suspected to present aTB were prospectively included when they first presented at the hospital with symptoms, if they agreed to participate in the study. aTB was diagnosed based on microbiological proof for most patients, and 14 patients who were initially suspected to present aTB but had an alternative final diagnosis were excluded from the final analysis (Fig. 1). Patients with aTB were included to evaluate the performance of the index test to differentiate LTBI from aTB. Their blood samples were always drawn before or after no more than 5 days of treatment.

A QuantiFERON-TB Gold In-Tube (QFT) assay, which is not included for diagnosis or classification in Belgium, was nevertheless performed for 72% of the LTBI subjects in the training cohort and, for research purposes, for all included subjects in the validation cohort. The QFT was performed on the same blood sample as the HBHA-IGRA and ESAT-6-IGRA for all of the samples from the validation cohort and for one-half of the tested samples from the training cohort.

### Ethical approval.

This study was approved by the Ethics Committee of ULB-Hôpital Erasme (aggregation number OMO21, study protocol P2007/175), and all of the patients and control subjects gave their written informed consent.

### IGRAs performed on PBMC.

The PBMC-HBHA-IGRA was performed as reported previously, using 2 μg/mL HBHA for *in vitro* stimulation of the PBMC (10), and a similar method was used for the PBMC-ESAT-6-IGRA, except that HBHA was replaced by 5 μg/mL recombinant ESAT-6 (Lionex, Braunschweig, Germany). A homemade PPD-IGRA using 4 μg/mL PPD (Statens Serum Institute, Copenhagen, Denmark) was also used to detect previous mycobacterial exposure. PBMC-IGRAs have been previously validated (11) and are therefore considered a reference for the evaluation of the new WB-IGRA in this study.

### WB-IGRAs.

The WB-IGRA was adapted from the standardized 24-h IGRAs performed on PBMC (11). Briefly, 250 μl WB was diluted 1:1 in interleukin 7 (IL-7)-enriched AIMV medium and incubated with the antigens for 24 h before supernatant collection (see the supplemental material for details). A mixture without antigen and another with staphylococcal enterotoxin B (SEB) (Sigma-Aldrich, Bornem, Belgium) were incubated as negative and positive controls, respectively. The optimal antigen concentrations were determined to be 4 μg/mL for HBHA and 5 μg/mL for ESAT-6 (see Fig. S1 in the supplemental material). IFN-γ concentrations were measured in the supernatants by a sandwich enzyme-linked immunosorbent assay (ELISA) according to the manufacturer’s instructions (ELISA IFN-γ Cytoset; Life Technologies, Ghent, Belgium). The positivity limit was determined by receiver operating characteristic curves established with results from LTBI subjects and noninfected controls (see Fig. S2). To take into account the inherent variability of the ELISA results, a gray zone of doubtful results was defined as 20% around the positivity limit of the test (see the supplemental material). The reproducibility of the WB-IGRA was evaluated by serial testing of blood samples collected from the same individuals (LTBI subjects and controls) at different time points (see Fig. S2).

### Statistical analyses.

GraphPad Prism v7.03 (GraphPad Software, La Jolla, CA, USA) was used for statistical analysis. The Kruskal-Wallis test followed by Dunn's multiple-comparison test was applied to compare continuous variables between the groups, while a chi-square test and Fisher’s exact test were used for categorical variables. Single comparisons of independent groups were performed using the Mann-Whitney test. Correlations were evaluated by a nonparametric Spearman test, and the degree of agreement between two tests was assessed by Cohen’s kappa coefficient. *P* values of <0.05 were considered significant.

### Data availability.

The data sets generated and/or analyzed in the current study are available from the corresponding author on request.

## RESULTS

### Study population characteristics.

Results from 83 subjects (20 controls, 32 with LTBI, and 31 with aTB) in the training cohort and from 69 subjects (25 controls, 27 with LTBI, and 17 with aTB) in the validation cohort were considered for the analysis of the IGRA results. The main demographic and clinical data for these subjects are reported in Table 1. The proportion of subjects originating from countries with endemicity was significantly smaller (*P* ≤ 0.05) for controls than for infected individuals and was the greatest among aTB patients. In both cohorts, most LTBI subjects were recruited among HCWs and HHCs with a remote infection (>2 years), as estimated by the time interval between TST positivity and blood sampling. None of these LTBI subjects progressed to TB disease. Only a minority of LTBI subjects received prophylactic anti-TB treatment before their inclusion in the study (Table 1).

**TABLE 1:** Characteristics of subjects with known Mycobacterium tuberculosis infection status

Parameter	Noninfected controls		LTBI subjects		aTB patients	
	Training cohort	Validation cohort	Training cohort	Validation cohort	Training Cohort	Validation cohort
No. of subjects	20	25	32	27	31	17
Age (median [range]) (yr)	33 (21–60)	43 (21–61)^*a*^	34 (21–64)	49 (19–64)^*b*^	33 (16–81)	40 (18–63)
Male/female (no./no.)	7/13	5/20	10/22	12/15	19/12^*c*^	9/7^*d*^
Ethnic origin (no. [%])						
Western Europe	15 (75)	24 (96)	13 (40)	14 (52)	4 (13)	0
Eastern Europe	1 (5)	0	6 (19)	2 (7)	5 (16)	3 (18)
North African	3 (15)	1 (4)	6 (19)	4 (15)	12 (39)	7 (41)
Central African	1 (5)	0	6 (19)	6 (22)	9 (29)	6 (35)
Other	0	0	1 (3)	1 (4)	1 (3)	1 (6)
M. bovis BCG vaccination status (no. [%])						
Vaccinated	6 (30)	5 (20)	22 (69)	19 (70)	4 (13)	1 (6)
Unvaccinated	12 (60)	16 (64)	7 (22)	7 (26)	6 (19)	3 (18)
Unknown	2 (10)	4 (16)	3 (9)	1 (4)	21 (68)	13 (76)
Possible M. tuberculosis exposure (no. [%])						
HHC	0	0	11 (35)	8 (30)	5 (16)	3 (18)
HCW	9 (45)	21 (84)	19 (59)	15 (56)	1 (3)	1 (6)
Country of endemicity (origin and/or travel)	0	2 (8)	2 (6)	2 (7)	22 (71)	13 (76)
No known M. tuberculosis exposure	11 (55)	2 (8)	0	2 (7)	3 (10)	0
TST results						
Induration diam (median [range]) (mm)			19 (10–40)	16 (10–25)	20 (15–30)	
<10 mm (no. [%])	20 (100)	25 (100)	0	0	0	
10–14 mm (no. [%])	0	0	6 (19)^*e*^	6 (27)^*f*^	0	
≥15 mm (no. [%])	0	0	26 (81)	16 (73)	8 (100)	
QFT results						
Positive (no./total no. tested [%])		0	7/23 (30)	8/27 (30)	6/8 (76)	15/17 (88)
Negative (no./total no. tested [%])		23/23 (100)	16/23 (70)	18/27 (66)	1/8 (12)	2/17 (12)
Indeterminate (no./total no. tested [%])		0	0	1/27 (4)	1/8 (12)	0
Unknown (no. [%])	20	2	9	0	23	0
LTBI characteristics (no. [%])						
Date of infection						
<2 yr			8 (25)	2 (7)		
>2 yr			19 (59)	20 (74)		
Unknown			5 (16)	5 (19)		
Past treatment						
Yes			7 (22)	4 (15)		
No			23 (72)	23 (85)		
Unknown			2 (6)	0		
TB characteristics (no. [%])						
Type						
Pulmonary					15 (48)	12 (71)
Extrapulmonary					16 (52)	5 (29)
Diagnosis						
Sputum/culture/PCR positive					28 (90)	16 (94)
Response to treatment					3 (10)	1 (6)

a *P* = 0.0161 versus noninfected controls from the training cohort.

b *P* = 0.0121 versus LTBI subjects from the training cohort.

c *P* = 0.0234 versus LTBI subjects from the same cohort.

d *P* = 0.0229 versus controls from the same cohort.

e Two of 6 were HCWs with TST conversion, and 4/6 were HHCs (1 with TST conversion and 3 with past infections).

f Three of 6 were HCWs with TST conversion, and 3/6 were HHCs with a history of infection.

### Validation of the 24-h WB-HBHA-IGRA and WB-ESAT-6-IGRA.

**FIG 2:**
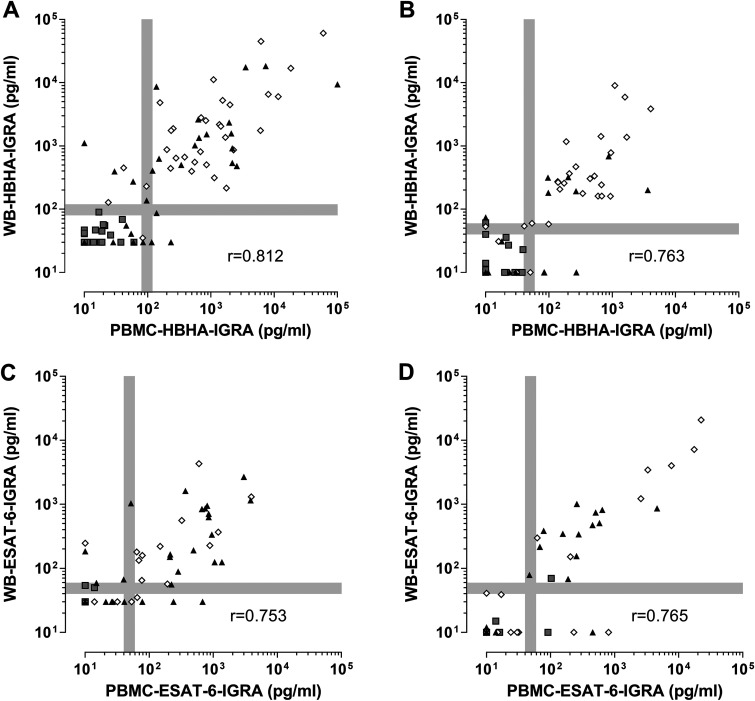
Correlations between the WB-IGRA and the PBMC-IGRA performed with HBHA or ESAT-6. PBMC or 2-fold diluted WB from the subjects of the training cohort (A and C) and the validation cohort (B and D) was stimulated for 24 h with 2 μg/mL HBHA for the PBMC-IGRA or 4 μg/mL HBHA for the WB-IGRA (A and B) or with 5 μg/mL ESAT-6 (C and D) before supernatant collection and IFN-γ concentration measurements. The IFN-γ concentrations obtained for the WB-IGRA and the PBMC-IGRA are represented as gray squares for noninfected controls, white diamonds for LTBI subjects, and black triangles for aTB patients. The Spearman’s rank correlation coefficients are indicated. The gray lines represent the gray zones corresponding to 20% variability around the cutoff values.

The WB-IGRAs were performed side by side with the IGRA performed on PBMC, and both the HBHA-induced and ESAT-6-induced IFN-γ levels were strongly correlated between the WB and PBMC in the two cohorts (Fig. 2). The degree of agreement between undoubtful results obtained by the two tests was very high (kappa values of 0.833 and 0.840 for HBHA and of 0.801 and 0.865 for ESAT-6 in the training and validation cohorts, respectively).

Using the WB-HBHA-IGRA and WB-ESAT-6-IGRA results, receiver operating characteristic curves for LTBI subjects in comparison with noninfected controls provided excellent area under the curve (AUC) values for HBHA, whereas those for aTB patients in comparison with noninfected controls provided better AUC values for ESAT-6 than for HBHA, indicating the superiority of the HBHA-IGRA to detect LTBI and of the ESAT-6-IGRA to detect aTB (see Fig. S2 in the supplemental material).

### Improved performance of the WB-HBHA-IGRA over the QFT to detect LTBI.

**FIG 3:**
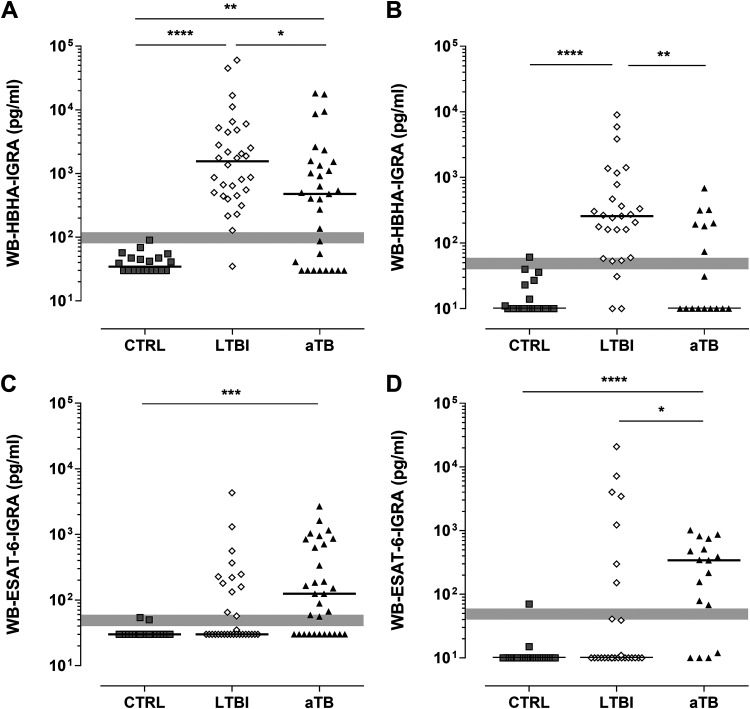
WB-HBHA-IGRA and WB-ESAT-6-IGRA results according to M. tuberculosis infection status. Twofold-diluted WB from the subjects in the training cohort (A and C) and the validation cohort (B and D) was stimulated for 24 h with 4 μg/mL HBHA (A and B) or 5 μg/mL ESAT-6 (C and D) before supernatant collection and IFN-γ concentration measurements. The IFN-γ concentrations obtained for the WB-IGRA are shown for noninfected controls (CTRL), LTBI subjects (LTBI), and aTB patients (aTB). The gray lines represent the gray zones corresponding to 20% variability around the cutoff values. For each group, the horizontal line represents the median of the results. *, *P* < 0.05; **, *P* < 0.01; ***, *P* < 0.001; ****, *P* < 0.0001.

Using the WB-HBHA-IGRA, HBHA-induced IFN-γ concentrations were very high in both cohorts in LTBI subjects, compared to controls (*P* < 0.0001), whereas they were less frequently elevated for aTB patients. The differences between aTB patients and controls were significant only in the training cohort (*P* < 0.01) (Fig. 3A and B). The HBHA-induced IFN-γ concentrations were higher for LTBI patients than for aTB patients (*P* < 0.05 and *P* < 0.01 for the training and validation cohorts, respectively) (Fig. 3A and B). When all positive results, including those within the gray zone, were considered, the WB-HBHA-IGRA detected all except 1 LTBI subject in the training cohort (97% sensitivity) and all except 3 LTBI subjects in the validation cohort (89% sensitivity), resulting in a global sensitivity of 93% (Fig. 1 and 3). This sensitivity was substantially higher than that of the QFT, as only 30% of the LTBI subjects were QFT positive in both cohorts, based on the recommended cutoff value of 0.35 IU/mL (Fig. 1). The specificity of the WB-HBHA-IGRA was 95% and 92% for the training and validation cohorts, respectively, due to a few doubtful results for noninfected subjects (Fig. 1 and 3), whereas it was 100% for the QFT (Fig. 1B).

### Identification of two subgroups of LTBI subjects by combining the WB-HBHA-IGRA and WB-ESAT-6-IGRA.

In contrast to HBHA, the ESAT-6-induced-IFN-γ concentrations were preferentially elevated during aTB, with significant differences between patients and noninfected controls (*P* < 0.001 and *P* < 0.0001 for the training and validation cohorts, respectively) (Fig. 3C and D). WB-ESAT-6-IGRA results for LTBI subjects were similar to those obtained with the QFT. Approximately one-third of the LTBI subjects were positive by the WB-ESAT-6-IGRA (38% and 30% in the training and validation cohorts, respectively). Using the WB-ESAT-6-IGRA, no significant differences were noticed between LTBI subjects and controls (Fig. 3C and D).

**FIG 4:**
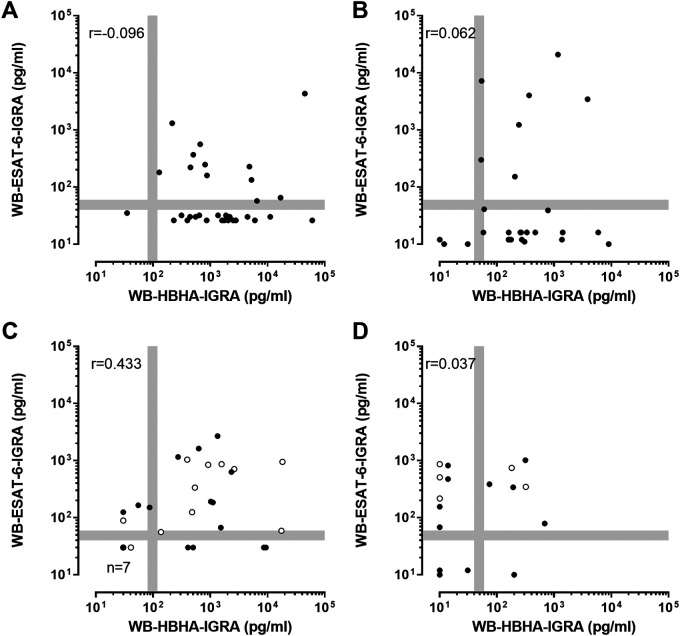
Correlations of the WB-HBHA-IGRA and WB-ESAT-6-IGRA results for LTBI subjects and aTB patients. Twofold-diluted WB from the subjects in the training cohort (A and C) and the validation cohort (B and D) was stimulated for 24 h with 4 μg/mL HBHA or 5 μg/mL ESAT-6 before supernatant collection and IFN-γ concentration measurements. The IFN-γ concentrations obtained with the WB-HBHA-IGRA and the WB-ESAT-6-IGRA are shown for LTBI subjects (A and B) and aTB patients (C and D). Patients with aTB had either pulmonary (black circles) or extrapulmonary (white circles) TB. The Spearman’s rank correlation coefficients are indicated. The gray lines represent the gray zones corresponding to 20% variability around the cutoff values.

Two LTBI subgroups could be identified by combining both WB-IGRAs. Most LTBI subjects were positive only for the WB-HBHA-IGRA, while some were positive for both (Fig. 4A and B). In contrast, aTB patients, including those with pulmonary and extrapulmonary aTB, were positive either for the WB-ESAT-6-IGRA or for both, whereas a positive WB-HBHA-IGRA result only was exceptional (Fig. 4C and D). No major demographic differences (age, sex, or country of origin) were observed between the two LTBI subgroups, and there was no difference in the proportions of subjects with recent/remote infections or TST sizes (Table 2). In contrast, WB-IGRA positivity with both antigens was more frequent among HHCs than among HCWs (*P* < 0.05 for the validation cohort and trend for the training cohort) (Table 2).

**TABLE 2:** Characteristics of the subgroups of LTBI subjects

Parameter	HBHA-IGRA-positive/ESAT-6-IGRA-negative		HBHA-IGRA-positive/ESAT-6-IGRA-positive	
	Training cohort	Validation cohort	Training cohort	Validation cohort
No. of subjects	19	16	12	8
Age (median [range]) (yr)	43 (22–64)	44 (32–64)	31 (21–45)	47 (19–55)
Male/female (no./no.)	7/12	5/11	3/9	5/3
Ethnic origin (no. [%])				
Western Europe	9 (47)	9 (57)	4 (33)	3 (38)
Eastern Europe	4 (21)	1 (6)	2 (17)	1 (12)
North African	4 (21)	1 (6)	2 (17)	3 (38)
Central African	2 (11)	5 (31)	3 (25)	0
Other	0	0	1 (8)	1 (12)
M. bovis BCG vaccination status (no. [%])				
Vaccinated	14 (74)	14 (88)	7 (58.3)	4 (50)
Unvaccinated	3 (15)	1 (6)	4 (33.3)	4 (50)
Unknown	2 (11)	1 (6)	1 (8.3)	0
Possible M. tuberculosis exposure (no. [%])				
HHC	4 (21)	2 (12.5)	7 (58)	5 (62.5)
HCW	14 (74)	11 (69)	5 (42)	2 (25)
Country of endemicity (origin and/or travel)	1 (5)	2 (12.5)	0	1 (12.5)
No known M. tuberculosis exposure	0	1 (6)	0	0
Date of infection (no. [%])				
<2 yr	5 (26)	1 (6)	3 (25)	0
>2 yr	14 (74)	14 (88)	5 (42)	4 (50)
Unknown	0	1 (6)	4 (33)	4 (50)
Treatment (no. [%])				
Yes	4 (21)	0	3 (25)	3 (38)
No	14 (74)	16 (100)	7 (58)	5 (62)
Unknown	1 (5)	0	2 (17)	0
TST results				
Induration diam (median [range]) (mm)	18 (10–40)	15 (10–25)	18 (13–28)	18 (15–22)
<10 mm (no. [%])	0	0	0	0
10–14 mm (no. [%])	4 (21)	0	2 (17)	0
≥15 mm (no. [%])	15 (79)	15 (100)	10 (83)	7 (100)

LTBI subjects identified by TST were thus easily differentiated from aTB patients, including those with pulmonary and extrapulmonary aTB, in case of a positive WB-HBHA-IGRA result only. The positive predictive values of a positive WB-HBHA-IGRA result only for LTBI, as opposed to aTB, were 83% and 94% in the training and validation cohorts, respectively. However, it remained difficult to differentiate LTBI from aTB when both WB-IGRA results were positive.

## DISCUSSION

Here, we report the development and validation of a new, user-friendly WB-IGRA based on 24-h *in vitro* stimulation of 1 mL blood (four 250-μl aliquots) with two mycobacterial antigens, HBHA and ESAT-6, in addition to negative and positive controls. We demonstrate in two independent cohorts the high sensitivity of the WB-HBHA-IGRA to detect LTBI subjects, in line with previous studies using a PBMC-HBHA-IGRA (10, 11, 13, 15). The WB-HBHA-IGRA was far more sensitive than the QFT to detect LTBI, providing 93% sensitivity with 93% specificity, compared to 30% sensitivity and 100% specificity for the QFT. By combining the WB-HBHA-IGRA and the WB-ESAT-6-IGRA, two different LTBI subpopulations were identified. A positive WB-HBHA-IGRA result only was characteristic of most LTBI patients and was exceptional among aTB patients, providing a clear differential diagnosis between LTBI and aTB. This might be particularly helpful to differentiate LTBI from extrapulmonary aTB, considering the difficulties of diagnosing extrapulmonary aTB. WB-IGRA positivity with both antigens was observed for some LTBI subjects and for aTB patients, in which case differential diagnosis remained difficult.

In several countries, it is now recommended to replace TST with commercial IGRAs for the detection of LTBI, especially among subjects with high Mycobacterium bovis bacillus Calmette-Guérin (BCG) vaccine coverage. However, various studies have shown discrepancies between TST and QFT results, which not only might be attributable to false-positive TST results but also were mostly a consequence of the lower sensitivity of the QFT, compared with the TST. A sensitivity of 30% was reported for the QFT among TST-positive subjects known to have been exposed to M. tuberculosis and living in the Netherlands, a country with low TB incidence (6). Similar results have been reported in Italy (7). In both studies, the sensitivity of commercial IGRAs could be improved by a longer *in vitro* incubation time (6 days versus 24 h), but 66% of the TST-positive subjects remained persistently IGRA negative (7). Two more recent larger studies came to the same conclusion of suboptimal sensitivity of the commercial ESAT-6 and CFP-10-based IGRAs to detect LTBI. The first study was performed with 5,357 adolescents in South Africa and reported that 68% of the adolescents with a negative QFT result (<0.35 IU/mL) had a positive TST result (18). The second study was performed in the Netherlands and evaluated 495 individuals who were suspected to have LTBI (19). Among them, only one-third had a positive QFT result, whereas 75% had a positive TST result. Efforts to reach higher sensitivity for LTBI diagnosis using the QFT, especially in immunocompromised subjects, involved the replacement of the QFT-GIT by the QFT-Plus. However, these two tests appear to perform equivalently for the detection of LTBI among immunocompetent individuals, except for elderly subjects (>75 years) (20, 21).

In this study, sensitivity for LTBI similar to that of the TST was reached by a 24-h WB-HBHA-IGRA. Combining the WB-HBHA-IGRA with the WB-ESAT-6-IGRA allows us to cover various stages that the mycobacteria may encounter during latency (2, 22). Whereas some LTBI subjects may harbor quiescent mycobacteria, others may harbor multiplying mycobacteria, maintained at a subclinical level, that express different antigens (22, 23). ESAT-6, which is also present in the commercial IGRAs, is highly expressed during bacterial multiplication (2, 24), while HBHA is a latency-associated antigen whose gene is upregulated under hypoxic conditions and in cells harboring M. tuberculosis during latency (24, 25). This approach has allowed us to identify two LTBI subgroups. LTBI subjects with an IFN-γ response to HBHA only likely harbor mostly quiescent bacteria and therefore may have a lower risk of reactivation. This is consistent with the notion that the IFN-γ response to HBHA has been proposed as a surrogate marker of protection (15). Studies in mice have indeed demonstrated that HBHA is a protective antigen against TB (26). In contrast to IFN-γ responses to HBHA only, the responses to both HBHA and ESAT-6 may reflect the simultaneous presence of quiescent and multiplying bacteria, suggesting a higher risk of TB reactivation, as illustrated by several case reports (27, 28). The proportion of such LTBI subjects, who thus also had a positive QFT result, was small in our study, because most LTBI subjects were in a long-term stage of latency, with a very low risk of reactivation. An IFN-γ response restricted to ESAT-6 was seen only in aTB, reflecting the presence of multiplying bacteria (24).

The strength of this study is the strict selection of uninfected controls and LTBI subjects, which allowed us to define the sensitivity and specificity of this novel IGRA. Controls with a positive PPD-IGRA result in spite of a negative TST result were excluded, to avoid the inclusion of possible TB resisters (17). LTBI subjects with an initial positive TST result, according to the Belgian TST-based criteria for LTBI detection (29), but with a negative PPD-IGRA result at the time of blood drawing were also excluded, because they might have cured their infection by the time of blood drawing. Most LTBI subjects were HCWs who were included more than 2 years after their identification as having LTBI via the TST. Thanks to a yearly medical follow-up assessment of HCWs, we could classify these subjects as real LTBI cases, because none of them developed aTB. Using these strict selection criteria, the training and validation cohorts provided similar results in terms of sensitivity and specificity for LTBI detection. However, these strict selection criteria for the participants might also be seen as a limitation of the study, because this might result in a selection bias. This could explain the poor performance of QFT in this study, because being a HCW was the only risk factor for TB exposure for most of the enrolled subjects. Other limitations resulted from the relatively small numbers of individuals included in these two cohorts. Therefore, larger studies performed with less restricted populations should be performed to confirm the message. The lack of a commercialized WB-HBHA-IGRA used here limits standardization of the assay for larger studies. Interest in the HBHA-IGRA was reported independently by several groups, mostly as an option to differentiate LTBI from aTB (10–13, 15, 30–32). However, technical differences between those studies, including different sources of HBHA, make comparisons of the results difficult. This might be mitigated by using a standardized, commercial WB-HBHA-IGRA, as recently stressed (32).

In conclusion, the WB-HBHA-IGRA and WB-ESAT-6-IGRA described here may potentially replace the TST, which is still used in several countries for LTBI detection. In addition to high sensitivity for LTBI, it may also help to differentiate LTBI from aTB (both pulmonary and extrapulmonary) and to identify LTBI subgroups, potentially leading to risk stratification. Therefore, the combined WB-IGRA represents an interesting tool for the screening of patients prior to immunosuppressive treatment, who are known to be at risk of TB reactivation. It may thereby help to prioritize patients for prophylactic anti-TB treatment, who should be those with dual responses to HBHA and ESAT-6.
