# Supplementary material for: Optimal Detection of Latent Mycobacterium tuberculosis Infection by Combined Heparin-Binding Hemagglutinin (HBHA) and Early Secreted Antigenic Target 6 (ESAT-6) Whole-Blood Interferon Gamma Release Assays
Source: J Clin Microbiol. 2022 Apr 18;60(5):e02443-21. doi: 10.1128/jcm.02443-21 (PMC9116186; doi:10.1128/jcm.02443-21)
Supplement: Supplemental file 1 — Text S1. Download jcm.02443-21-s0001.pdf, PDF file, 0.08 MB [file jcm.02443-21-s0001.pdf]

Supplementary material

Whole blood interferon-γ-release assay

Whole blood was collected by venipuncture using BD Vacutainer Sodium Heparin Tubes (BD Biosciences, Erembodegem, Belgium) and sent to the laboratory at room temperature. Within 6 hrs after blood collection, one mL whole blood was diluted with a 1 mL of the serum-free medium AIMV (Thermo Fisher scientific - Life technologies Europe, Merelbeke, Belgium). Interleukin-7 (Biotechne - R&D Systems, Abingdon, United Kingdom) was added to the suspension at 1 ng/ml, as defined previously for the PBMC-IGRA [1]. Then, 500 µl of diluted blood was distributed in 5-ml round-bottom polypropylene tubes (BD Falcon, VWR International, Leuven, Belgium), corresponding to the stimulation of 250 µl original blood per tube. The diluted blood was stimulated with either (i) 4 µg/ml native HBHA, (ii) 5 µg/ml ESAT-6 (Lionex, Braunschweig, Germany), (iii) 1 µg/ml superantigen staphylococcal enterotoxin B (SEB) (Sigma-Aldrich, Bornem, Belgium) as a positive control, or (iv) an antigen-free IL7-enriched medium as a negative control. After 24 hrs incubation (range, 22hrs-26hrs) at 37°C (5% CO_2), the tubes were centrifuged at 500 x *g* during 5 min. and the culture supernatants were collected and stored at -20°C until measurement of IFN-γ concentrations. These were measured by classical sandwich enzyme-linked immunosorbent assay (ELISA) according to the manufacturer's instructions (ELISA IFN-γ Cytoset; Life Technologies, Ghent, Belgium). Control values of >200 pg/ml in response to SEB and <50 pg/ml in the negative control were required for further analysis. When detectable, the IFN-γ concentrations obtained under non-stimulated conditions were subtracted from those obtained for the stimulated conditions. Optimal cut-offs were determined by receiver operating characteristic curves performed with results from LTBI subjects and non-infected controls (Figure S2). A grey zone was defined as 20% around the cut-off values to take into account the ELISA coefficient of variation. Results within this grey zone were therefore considered as doubtful. Native HBHA was purified from

M. bovis BCG (strain 1173P2; World Health Organization) grown in static cultures at 37°C and then centrifuged, heat-inactivated and sonicated, as described [2]. HBHA was then purified by heparin-Sepharose chromatography or SP-Sepharose, followed by reverse-phase high pressure liquid chromatography, as described [3]. Recombinant ESAT-6 was provided by Lionex (Braunschweig, Germany).

[1] Wyndham-Thomas C, Corbière V, Dirix V, Smits K, Domont F, Libin M et al. Key role of effector memory CD4+ T lymphocytes in short-incubation heparin-binding hemagglutinin gamma interferon release assay for the detection of latent tuberculosis. Clin Vaccine Immunol 2014; 21(3): 321- 8.

[2] Menozzi FD, Rouse JH, Alavi M, Laude-Sharp M, Muller J, Bischoff R, et al. Identification of a heparin-binding hemagglutinin present in mycobacteria. J Exp Med 1996; 184:993–1001

[3] Corbière V, Segers J, Desmet R, Lecher S, Loyens M, Petit E et al. Natural T Cell Epitope Containing Methyl Lysines on Mycobacterial Heparin-Binding Hemagglutinin. J Immunol. 2020;204(7):1715-23.
