# Supplementary material for: Optimal Detection of Latent Mycobacterium tuberculosis Infection by Combined Heparin-Binding Hemagglutinin (HBHA) and Early Secreted Antigenic Target 6 (ESAT-6) Whole-Blood Interferon Gamma Release Assays
Source: J Clin Microbiol. 2022 Apr 18;60(5):e02443-21. doi: 10.1128/jcm.02443-21 (PMC9116186; doi:10.1128/jcm.02443-21)
Supplement: Supplemental file 2 — Table S1. Download jcm.02443-21-s0002.pdf, PDF file, 0.07 MB [file jcm.02443-21-s0002.pdf]

Supplementary Table

Table S1: Criteria for the classification of the individuals as non-infected controls, LTBI subjects, aTB patients.

	**Symptoms**	**TST size induration ***	**PPD-IGRA$**	**Chest radiograph**	**Microbiology**
**Non-infected controls**	Absent	Negative during last 24 months	Negative	ND	ND
**LTBI subjects**					
* *M. tuberculosis* exposure risk ++	Absent	≥ 10 mm	Positive	Normal	ND
* M. tuberculosis exposure risk ±	Absent	≥ 15 mm or TST conversion **	Positive	Normal	ND
**aTB** (untreated or treated for less than 5 days)	Present/ absent	ND	Positive	Normal/ abnormal	Positive

LTBI, latent tuberculosis infection; aTB, active TB; TST, Tuberculin Skin Test; PPD, purified protein derivative; IGRA IFN-γ release assay; ND not done

*TST performed by the intradermal injection of two IU of PPD RT23 (tuberculin purified protein derivative, Statens Serum Institute, Copenhagen, Denmark) with measurement of the induration size 72 hrs later
**TST conversion: change of at least 10 mm in the size of induration between two TST
$ performed at inclusion on PBMC as described in Wyndham-Thomas et al. [1]

[1] Wyndham-Thomas C, Corbière V, Dirix V, Smits K, Domont F, Libin M et al. Key role of effector memory CD4+ T lymphocytes in short-incubation heparin-binding hemagglutinin gamma interferon release assay for the detection of latent tuberculosis. Clin Vaccine Immunol 2014; 21(3): 321- 8.
