# Supplementary material for: Optimal Detection of Latent Mycobacterium tuberculosis Infection by Combined Heparin-Binding Hemagglutinin (HBHA) and Early Secreted Antigenic Target 6 (ESAT-6) Whole-Blood Interferon Gamma Release Assays
Source: J Clin Microbiol. 2022 Apr 18;60(5):e02443-21. doi: 10.1128/jcm.02443-21 (PMC9116186; doi:10.1128/jcm.02443-21)
Supplement: Supplemental file 3 — Fig. S1. Download jcm.02443-21-s0003.pdf, PDF file, 0.2 MB [file jcm.02443-21-s0003.pdf]

Supplementary Figure 1

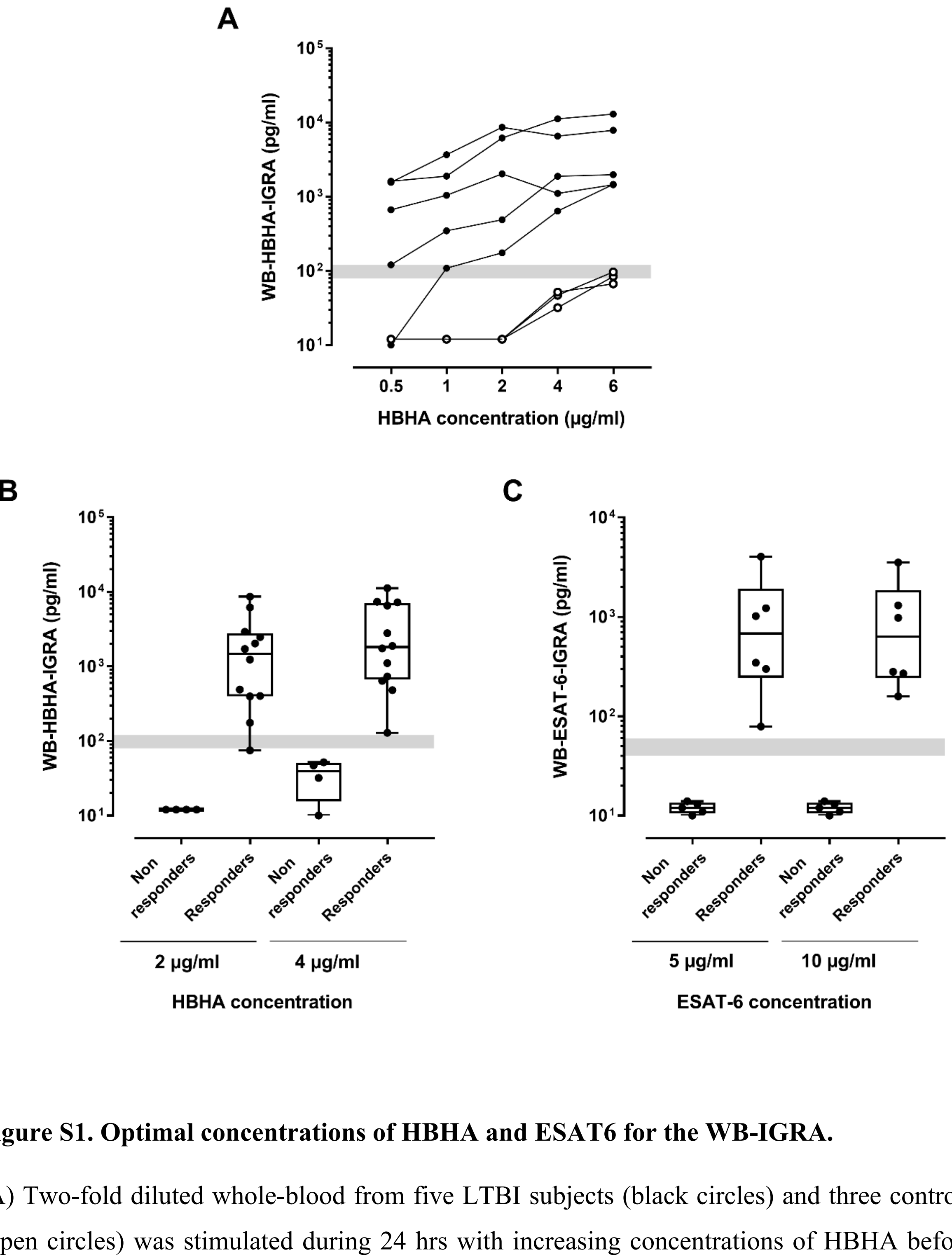

Figure S1. Optimal concentrations of HBHA and ESAT6 for the WB-IGRA.

(A) Two-fold diluted whole-blood from five LTBI subjects (black circles) and three controls (open circles) was stimulated during 24 hrs with increasing concentrations of HBHA before supernatant collection and IFN-γ concentration measurements. (B) Two-fold diluted whole-blood from nine LTBI subjects, three aTB patients and four controls was stimulated during 24 hrs with HBHA at 2 µg/ml or 4 µg/ml before supernatant collection and IFN-γ concentration

measurements. Results are depicted as medians (horizontal bars), 25th–75th percentiles (boxes) and ranges, together with all data points. (C) Two-fold whole-blood from six LTBI subjects, five aTB patients and one control were stimulated during 24 hrs with ESAT-6 at 5 μg/ml or 10 μg/ml before supernatant collection and IFN-γ concentration measurements. Results are depicted as medians (horizontal bars), 25th–75th percentiles (boxes) and ranges, together with all data points. The grey lines represent the grey zones corresponding to 20% variability around the cut-off.
