# Supplementary material for: Optimal Detection of Latent Mycobacterium tuberculosis Infection by Combined Heparin-Binding Hemagglutinin (HBHA) and Early Secreted Antigenic Target 6 (ESAT-6) Whole-Blood Interferon Gamma Release Assays
Source: J Clin Microbiol. 2022 Apr 18;60(5):e02443-21. doi: 10.1128/jcm.02443-21 (PMC9116186; doi:10.1128/jcm.02443-21)
Supplement: Supplemental file 4 — Fig. S2. Download jcm.02443-21-s0004.pdf, PDF file, 0.2 MB [file jcm.02443-21-s0004.pdf]

Supplementary Figure 2

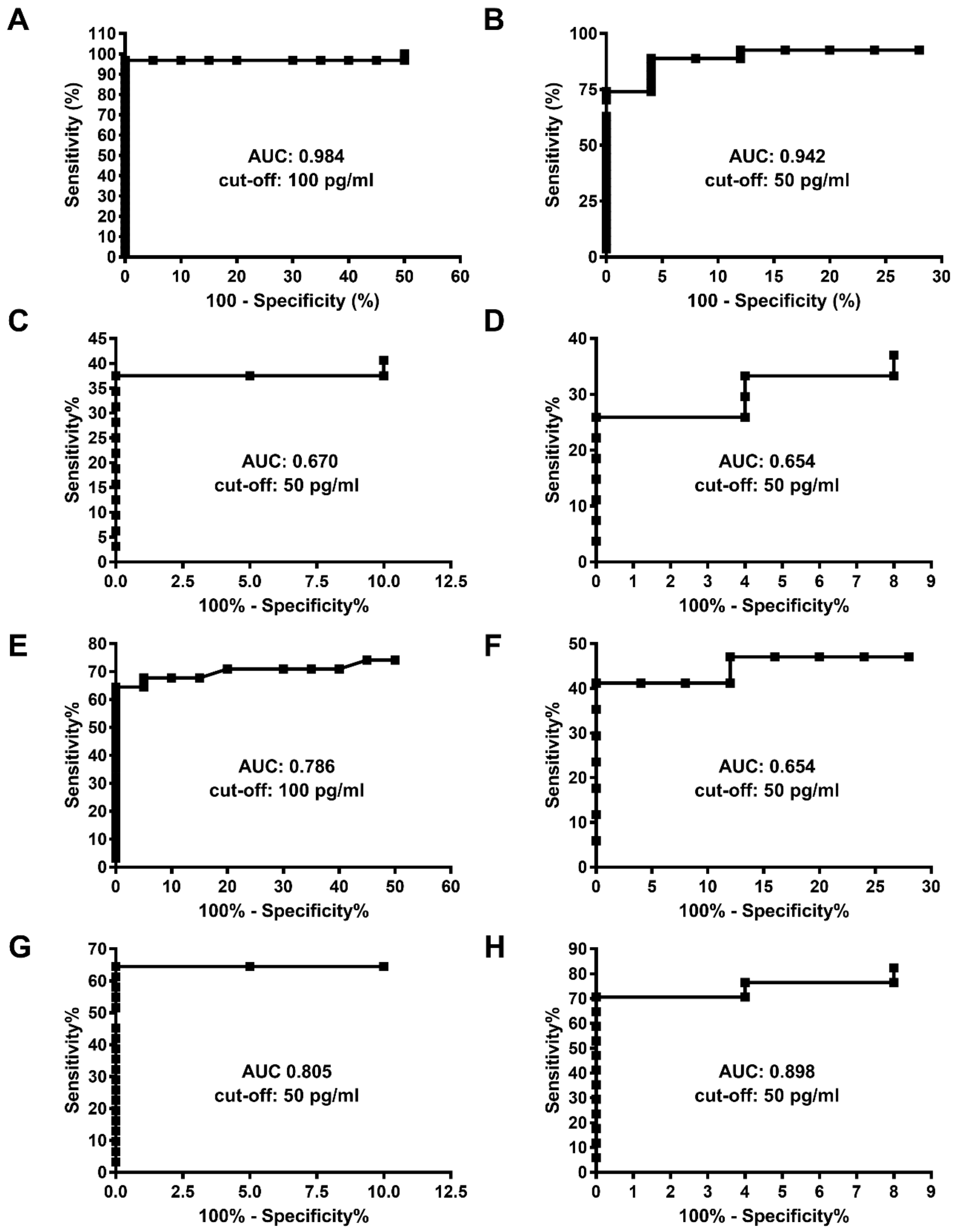

Figure S2. Receiver operating characteristic curve analysis of the WB-HBHA- and WB-ESAT-6-IGRA in controls, LTBI subjects and aTB patients.

Two-fold diluted whole-blood from the subjects of the training cohort (A, C, E, G) and the validation cohort (B, D, F, G) were stimulated during 24 hrs with 4 µg/ml HBHA (A, B, E, F) or 5 µg/ml ESAT-6 (C, D, G, H) before supernatant collection and IFN-γ concentration measurements. ROC curves were established for each antigen for LTBI subjects compared to controls (A, B, C, D) and for aTB patients compared to controls (E, F, G, H). The Area Under the curves (AUC) and the cut-offs chosen to provide the best sensitivity and specificity for LTBI-CTRL are indicated.
