# Supplementary material for: Optimal Detection of Latent Mycobacterium tuberculosis Infection by Combined Heparin-Binding Hemagglutinin (HBHA) and Early Secreted Antigenic Target 6 (ESAT-6) Whole-Blood Interferon Gamma Release Assays
Source: J Clin Microbiol. 2022 Apr 18;60(5):e02443-21. doi: 10.1128/jcm.02443-21 (PMC9116186; doi:10.1128/jcm.02443-21)
Supplement: Supplemental file 5 — Fig. S3. Download jcm.02443-21-s0005.pdf, PDF file, 0.2 MB [file jcm.02443-21-s0005.pdf]

Supplementary Figure 3

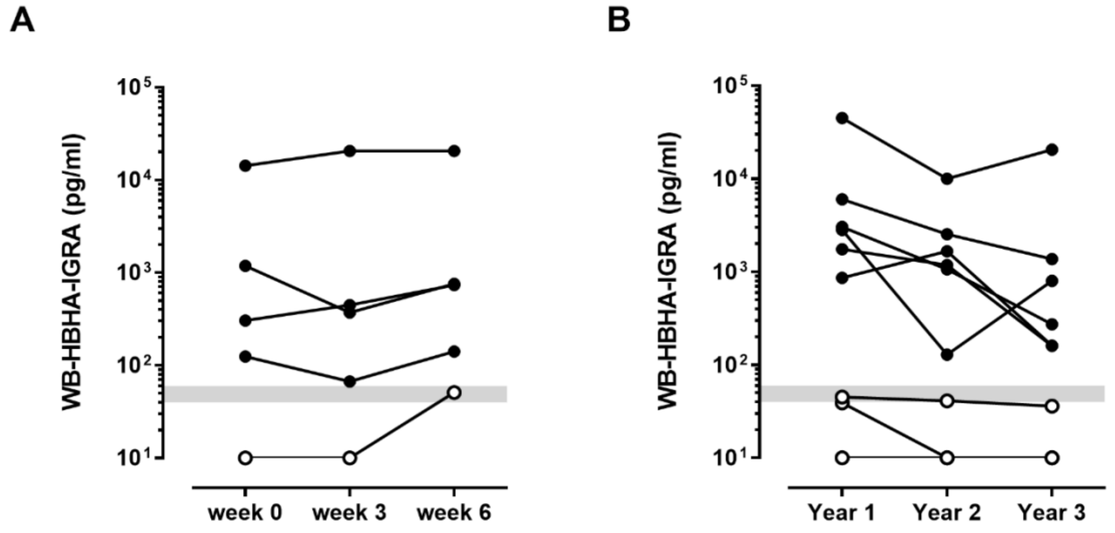

Figure S2. Reproducibility of the WB-HBHA-IGRA.

(A) The reproducibility of the WB-HBHA-IGRA was evaluated on a 3-weeks interval blood collection from four LTBI subjects (black circles) and one control (open circle). At each time point, two-fold diluted whole-blood was stimulated during 24 hrs with 4µg/ml HBHA before supernatant collection and IFN-γ concentration measurements. (B) The reproducibility of the WB-HBHA-IGRA was evaluated on a 3-years interval blood collection from six LTBI subjects (black circles) and three controls (open circles). At each time point, two-fold diluted whole-blood from was stimulated during 24 hrs with 4 µg/ml HBHA before supernatant collection and IFN-γ concentration measurements. The grey lines represent the grey zones corresponding to 20% variability around the cut-off.
